# Supplementary material for: The Effectiveness and Sero-Immunity of Hepatitis B Vaccination in People Who Use Drugs: A Systematic Review and Meta-Analysis
Source: Vaccines (Basel). 2024 Sep 8;12(9):1026. doi: 10.3390/vaccines12091026 (PMC11435961; doi:10.3390/vaccines12091026)
Supplement: Supplementary file 1 [file vaccines-12-01026-s001.zip › vaccines-3160719-supplementary.pdf]

Supplementary Materials:

Figure S1. Search strategy in Pubmed

Pubmed: All fields

"Hepatitis B*" OR "Hepatitis B"[Mesh]

AND

“Vaccin*” OR "Immunization, Secondary*" OR "Immunization, Secondary"[Mesh] OR "Vaccination in infancy" OR "primary vaccin*"

AND

"Breakthrough Infection" OR ("Breakthrough Infections"[Mesh]) OR “antigens, hepatitis b” OR ("Hepatitis B Antigens"[Mesh]) OR "Long-term Immunity" OR "hepatitis B infection*" OR "Hepatitis B, Chronic" OR ("Hepatitis B, Chronic"[Mesh]) OR "Anamnestic response" OR "Long-term" OR "Immunologic Memory" OR immun* OR "immune coverage" OR "immune persistence" OR booster* OR Protect* OR "HBs Ag" OR "HBsAg" OR "AntiHBc" OR "Anti HBc" OR "hepatitis B core antibody" OR "hepatitis B surface antigen" OR ("Hepatitis B Surface Antigens"[Mesh])

AND

Incid* OR ("Incidence"[Mesh]) OR Prevalen* OR ("Prevalence"[Mesh])

Figure S2. Search strategy in Scopus

Scopus : Article title, Abstract, Keywords

"Hepatitis B*"

AND

“Vaccin*” OR "Immunization, Secondary*" OR "Vaccination in infancy" OR "primary vaccin*"

AND

"Breakthrough Infection" OR “antigens, hepatitis b” OR "Long-term Immunity" OR "hepatitis B infection*" OR "Hepatitis B, Chronic" OR "Anamnestic response" OR "Long-term" OR "Immunologic Memory" OR immun* OR "immune coverage" OR "immune persistence" OR booster* OR Protect* OR "HBs Ag" OR "HBsAg" OR "AntiHBc" OR "Anti HBc" OR "hepatitis B core antibody" OR "hepatitis B surface antigen"

AND

(Incid* OR Prevalen*)

Figure S3. Search strategy in Web of Science

Web Of Science : All fields

"Hepatitis B*"

AND

“Vaccin*” OR "Immunization, Secondary*" OR "Vaccination in infancy" OR "primary vaccin*"

AND

"Breakthrough Infection" OR “antigens, hepatitis b” OR "Long-term Immunity" OR "hepatitis B infection*" OR "Hepatitis B, Chronic" OR "Anamnestic response" OR "Long-term" OR "Immunologic Memory" OR immun* OR "immune coverage" OR "immune persistence" OR booster* OR Protect* OR "HBs Ag" OR "HBsAg" OR "AntiHBc" OR "Anti HBc" OR "hepatitis B core antibody" OR "hepatitis B surface antigen"

AND

(Incid* OR Prevalen*)

Figure S4. Search strategy in Cochrane Library

Cochrane Library : All text

Hepatitis NEXT B*

AND

(NEXT Vaccin*) OR (Immunization, NEXT Secondary*) OR "Vaccination in infancy" OR (primary NEXT vaccin*)

AND

"Breakthrough Infection" OR “antigens, hepatitis b” OR "Long-term Immunity" OR (hepatitis B NEXT infection*) OR "Hepatitis B, Chronic" OR "Anamnestic response" OR "Long-term" OR "Immunologic Memory" OR (NEXT immun*) OR "immune coverage" OR "immune persistence" OR (NEXT booster*) OR Protect* OR "HBs Ag" OR "HBsAg" OR "AntiHBc" OR "Anti HBc" OR "hepatitis B core antibody" OR "hepatitis B surface antigen"

AND

(NEXT Incid*) OR (NEXT Prevalen*)

Figure S5. Funnel plots of publications regarding effectiveness and sero-immunity of Hepatitis B Vaccination in People Who Use Drugs.

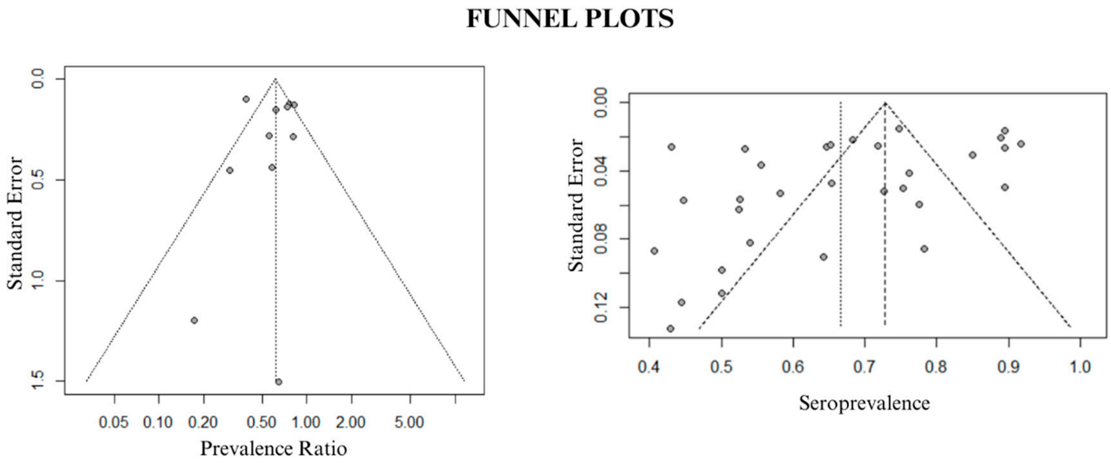

(a) Occurrence of infection **(b)** Seroimmunity

Figure S6. Vaccine effectiveness measured as positive HBsAg in People Who Use Drugs by Diagnosis Test.

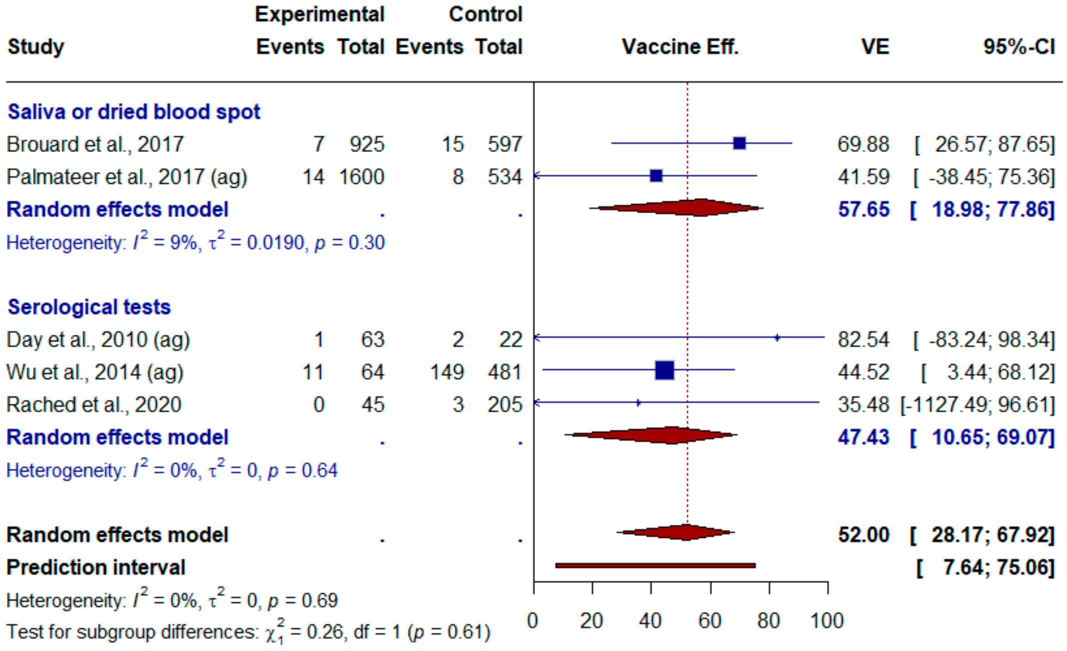

Figure S7. Vaccine effectiveness measured as positive anti-Hbc in People Who Use Drugs by Diagnosis Test.

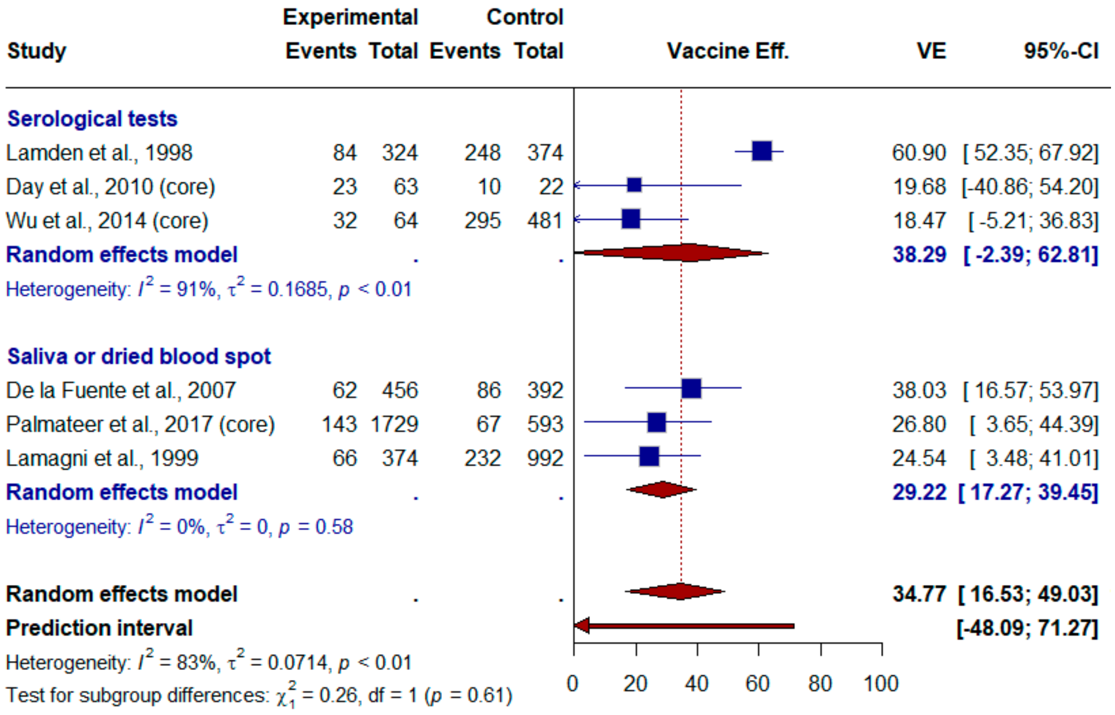

Figure S8. Vaccine effectiveness measured as positive HBsAg in People Who Use Drugs by Age.

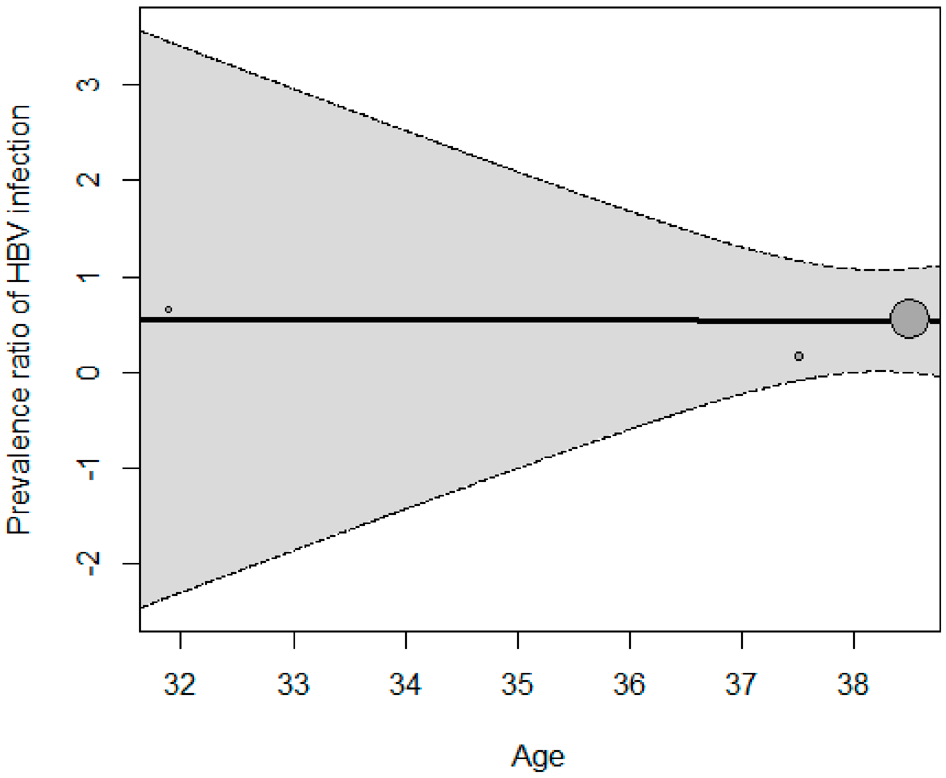

Figure S9. Vaccine effectiveness measured as positive anti-Hbc in People Who Use Drugs by Age.

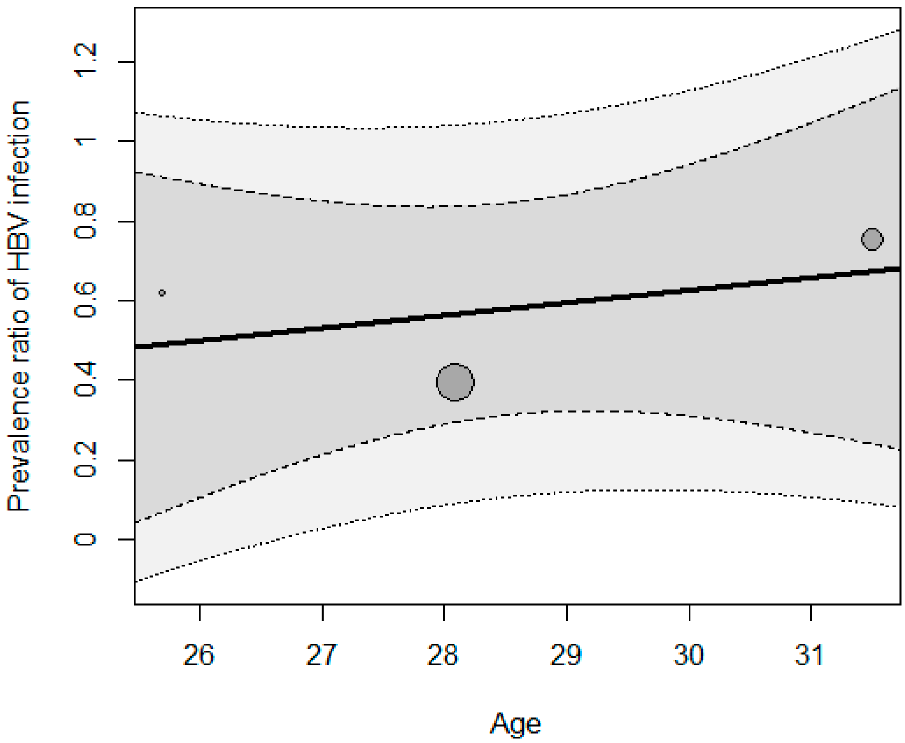

Figure S10. Prevalence of sero-immunity (anti-Hbs) in People Who Use Drugs.

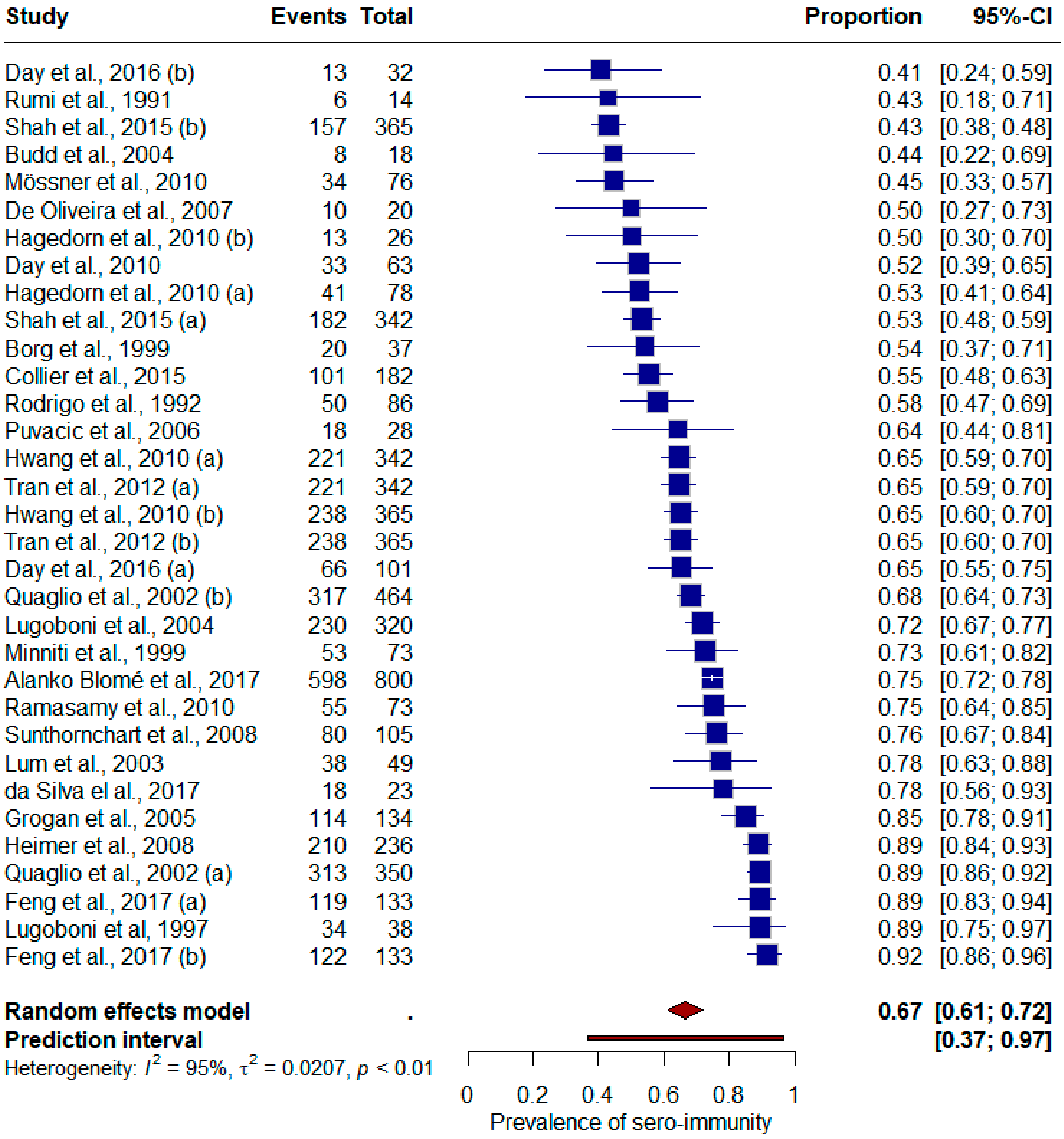

Figure S11. Prevalence of sero-immunity (anti-Hbs) in People Who Use Drugs by vaccination schedule.

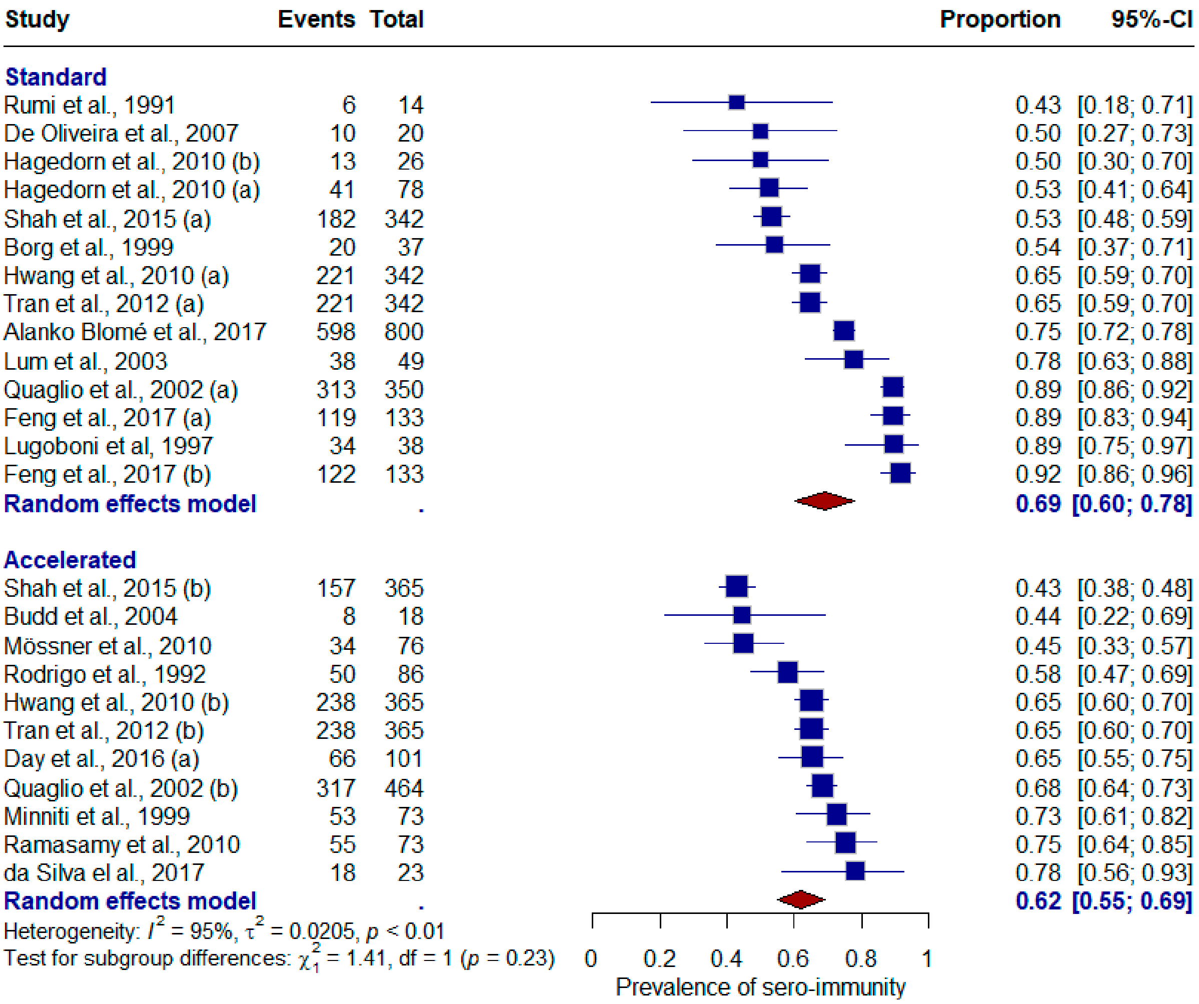

Figure S12. Prevalence of sero-immunity (anti-Hbs) in People Who Use Drugs by Age

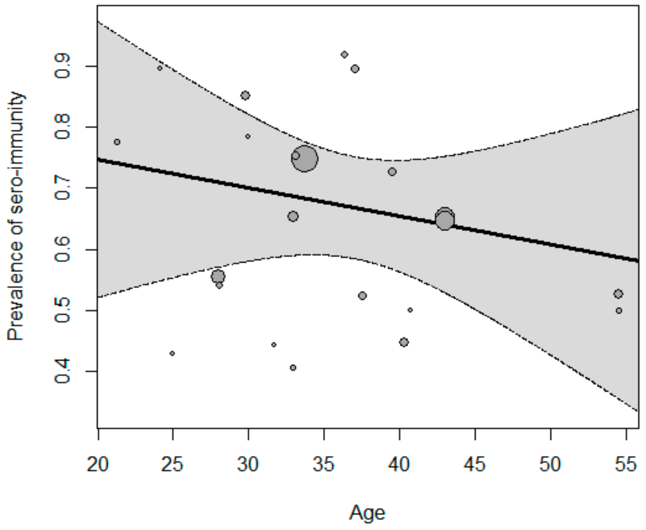

Table S1. Excluded articles and criteria for exclusion.

Author	Motivo de exclusión
Amesty et al., 2008	No vaccination data
Anderson et al., 1994	No vaccination data
Andersson et al., 2012	Data not extractable
Borovčanin et al., 2019	Data not extractable
Burt et al., 2007	Data not extractable
Craine et al., 2004	Data not extractable
Delgado-Iribarren et al., 2000	No vaccination data
Dong et al., 2015	No vaccination data
Edeh et al., 2000	Data not extractable
Ferreira el al., 2009	No vaccination data
Figgatt et al., 2020	Data not extractable
Fitzgerald et al., 2001	Data not extractable
Gerlich et al., 2006	Data not extractable
Gunn et al., 2005	Data not extractable
Haussig et al., 2018	Data not extractable
Hope et al., 2016	Data not extractable
Kandelouei et al., 2013	Data not extractable
Keten et al., 2016	No vaccination data
Khodadoostan et al., 2014	No vaccination data
Kilonzo et al., 2021	Data not extractable
Koene et al., 2009	Infected prior to vaccination
Kolovrat et al., 2010	Data not extractable
Kunches et al., 2016	No data on serological test and/or interpretation
Kuo et al., 2004	No vaccination data
Kuo et al., 2004	No data on serological test and/or interpretation
Legoupil et al., 2017	Data not extractable
Lugoboni et al., 2015	No vaccination data
Marchesini et al., 2007	Data not extractable
Neaigus et al., 2007	No vaccination data
Njoroge et al., 2019	Data not extractable
Osimani et al., 2005	No vaccination data
Perlman et al., 2014	No data on serological test and/or interpretation
Piauiense et al., 2020	Data not extractable
Pinar et al., 2022	No vaccination data
Quan et al., 2009	No data on serological test and/or interpretation
Removille et al., 2011	Data not extractable
Roy et al., 2008	No vaccination data
Schalm et al., 1983	Data not extractable
Schreuder et al., 2010	Data not extractable
Shing et al., 2020	No vaccination data
Vallejo et al., 2008	No vaccination data
van Santen et al., 2021	No vaccination data
Winter et al., 2013	No vaccination data
Ximenes et al., 2015	Data not extractable
Yüncü et al., 2008	Data not extractable
Zocratto et al., 2010	No vaccination data

Table S2. Risk of Bias evaluation with ROBINS-E tool

	ROBINS-E RISK OF BIAS DOMAINS							OVERALL RISK OF BIAS
**Study**	**D1**	**D2**	**D3**	**D4**	**D5**	**D6**	**D7**	
Alanko Blomé et al., 2017	Low	Low	Low	Low	Low	Low	Low	Low
Brouard et al., 2017	Low	Some Concerns	Low	Low	Low	Low	Low	Some Concerns
Collier et al., 2015	Low	Some Concerns	Low	Low	Low	Low	Low	Some Concerns
da Silva el al., 2017	Low	Low	Low	Low	Some Concerns	Low	Low	Some Concerns
Day et al., 2010	Low	Some Concerns	Low	Low	Some Concerns	Low	Low	Some Concerns
De la Fuente et al., 2007	Some Concerns	Some Concerns	Low	Low	Low	Low	Low	Some Concerns
De Oliveira et al., 2007	Low	Low	Low	Low	Low	Low	Low	Low
Grogan et al., 2005	Some Concerns	Low	Low	Low	Low	Low	Low	Some Concerns
Hagedorn et al., 2010	Low	Low	Low	Low	Low	Low	Low	Low
Lamagni et al., 1999	Some Concerns	Some Concerns	Low	Low	Low	Low	Low	Some Concerns
Lamden et al., 1998	Low	Low	Low	Low	Some Concerns	Low	Low	Some Concerns
Lugoboni et al., 2004	Some Concerns	Low	Low	Low	Some Concerns	Low	Low	Some Concerns
Mössner et al., 2010	Some Concerns	Low	Low	Low	Some Concerns	Low	Low	Some Concerns
Palmateer et al., 2017	Low	Some Concerns	Low	Low	Some Concerns	Low	Low	Some Concerns
Puvačić et al., 2006	Some Concerns	Some Concerns	Low	Low	Low	Low	Low	Some Concerns
Rached et al., 2020	Some Concerns	Some Concerns	Low	Low	Low	Low	Low	Some Concerns
Wu et al., 2014	Some Concerns	Some Concerns	Low	Low	Low	Low	Low	Some Concerns

Domains:
D1: Risk of bias due to confounding.
D2: Risk of bias arising from measurement of the exposure.
D3: Risk of bias in selection of participants into the study (or into the analysis).
D4: Risk of bias due to post-exposure interventions.
D5: Risk of bias due to missing data.
D6: Risk of bias arising from measurement of the outcome.
D7: Risk of bias in selection of the reported result.

Judgement:
- (green) ***Low Risk of Bias, except for concerns about residual confounding***
- (yellow) ***Some Concerns***
- (red) ***High Risk of Bias***
- (dark red) ***Very High Risk of Bias***

Table S3. Risk of Bias evaluation with ROBINS-I tool

	ROBINS-I RISK OF BIAS DOMAINS							OVERALL RISK OF BIAS
**Study**	**D1**	**D2**	**D3**	**D4**	**D5**	**D6**	**D7**	
Borg et al., 1999	🟢	🟢	🟢	🟢	🟡	🟢	🟢	🟢
Budd et al., 2004	🟢	🟢	🟢	🟡	🟡	🟢	🟢	🟡
Day et al., 2016	🟢	🟢	🟢	🟢	🟢	🟢	🟢	🟢
Feng et al., 2017	🟢	🟢	🟢	🟢	🟡	🟢	🟢	🟡
Heimer et al., 2008	🟢	🟢	🟢	🟢	🟢	🟢	🟢	🟢
Lugoboni et al., 1997	🟢	🟢	🟢	🟢	🟡	🟢	🟢	🟡
Hwang et al., 2010	🟢	🟢	🟢	🟢	🟢	🟢	🟢	🟢
Lum et al., 2003	🟢	🟢	🟢	🟡	🟡	🟢	🟢	🟢
Minniti et al., 1999	🟢	🟢	🟢	🟢	🟢	🟢	🟢	🟢
Quaglio et al., 2002	🟢	🟢	🟢	🟡	🟡	🟢	🟢	🟢
Ramasamy et al., 2010	🟢	🟢	🟢	🟡	🟡	🟢	🟢	🟡
Rodrigo et al., 1992	🟢	🟢	🟢	🟡	🟡	🟢	🟢	🟡
Rumi et al., 1991	🟢	🟢	🟢	🟢	🟡	🟢	🟢	🟡
Shah et al., 2015	🟢	🟢	🟢	🟡	🟡	🟢	🟢	🟡
Sunthornchart et al., 2008	🟢	🟢	🟢	🟡	🟡	🟢	🟢	🟡
Tran et al., 2012	🟢	🟢	🟢	🟢	🟢	🟢	🟢	🟢

Domains:

D1: Bias due to confounding.
D2: Bias in selection of participants into the study.
D3: Bias in classification of interventions.
D4: Bias due to deviations from intended interventions.
D5: Bias due to missing data.
D6: Bias in measurement of the outcomes.
D7: Bias in selection of the reported result.

Judgement:

🟢 ***Low Risk of Bias***
🟡 ***Moderate Risk of Bias***
🔴 ***Serious Risk of Bias***
🟤 ***Critical Risk of Bias***
